# Craniofacial and upper airway morphological characteristics associated with the presence and severity of obstructive sleep apnea in Chinese children

**DOI:** 10.3389/fped.2023.1124610

**Published:** 2023-03-31

**Authors:** Qiuping Xu, Xiaoya Wang, Na Li, Ying Wang, Xin Xu, Jing Guo

**Affiliations:** ^1^ Engineering Laboratory for Biomaterials and Tissue Regeneration, Ningbo Stomatology Hospital, Ningbo, Zhejiang, China & Savaid Stomatology School of Hangzhou Medical College, Hangzhou, Zhejiang, China; ^2^ Department of Orthodontics, School and Hospital of Stomatology, Cheeloo College of Medicine, Shandong University & Shandong Key Laboratory of Oral Tissue Regeneration & Shandong Engineering Laboratory for Dental Materials and Oral Tissue Regeneration & Shandong Provincial Clinical Research Center for Oral Diseases, Jinan, China; ^3^ Department of Stomatology, Beijing Tongren Hospital, Capital Medical University, Beijing, China; ^4^ Department of Implantology, School and Hospital of Stomatology, Cheeloo College of Medicine, Shandong University & Shandong Key Laboratory of Oral Tissue Regeneration & Shandong Engineering Laboratory for Dental Materials and Oral Tissue Regeneration & Shandong Provincial Clinical Research Center for Oral Diseases, Jinan, China

**Keywords:** orthodontic clamps, dentofacial orthopedics, upper airway, pediatric obstructive sleep apnea, cephalometry, craniofacial abnormalities

## Abstract

**Objectives:**

To identify craniofacial and upper airway morphological characteristics associated with the presence and severity of obstructive sleep apnea (OSA) in children.

**Methods:**

This study consisted of 82 OSA children and 77 controls (age 5–10 years). All subjects underwent cephalograms and were divided into a 5–7 age group and an 8–10 age group. Cephalometric variables were compared between OSA children and controls, and hierarchical regression analysis was performed to examine the relationship between cephalometric variables and OSA severity [expressed by the obstructive apnea–hypopnea index (OAHI)] in different age groups.

**Results:**

Increased A/N ratio, narrowed posterior airway space, decreased SNA and SNB angles, and shortened ramus height were observed among OSA children in different age groups. In the 5–7 age group, the A/N ratio and a lower gonial angle explained 40.0% and 14.7% of the variance in the OAHI, respectively. In the 8–10 age group, the BMI z-score and A/N ratio explained 25.2% and 6.6% of the variance in the OAHI, followed by a lower gonial angle and the hyoid-retrognathion distance (19.1% in total).

**Conclusions:**

Adenoid hypertrophy was a major factor associated with OSA in preschool children, whereas obesity replaced adenoid hypertrophy as the main contributor to OSA in late childhood. Several craniofacial skeletal variables such as the SNB angle, ramus height, lower gonial angle, and hyoid position are also associated with the presence and/or severity of OSA, which could be used to help recognize children at a higher risk for OSA.

## Introduction

Obstructive sleep apnea (OSA) is characterized by recurrent partial or complete upper airway obstruction during sleep that interrupts normal sleep patterns and ventilation, therefore resulting in intermittent hypoxemia, hypercarbia, and/or sleep fragmentation (1). OSA in children is associated with a number of significant complications, such as neurocognitive impairments, learning deficits, behavioral problems, growth retardation, pulmonary hypertension, and cardiac dysfunction (2–5). The early identification and proper treatment of pediatric OSA are essential to prevent these deleterious complications.

Adenotonsillar hypertrophy has been generally considered the most common risk factor for OSA in otherwise normal healthy children (6). Enlarged upper airway lymphoid tissues will increase pharyngeal resistance when superimposed with other functional factors (e.g., reduced neuromuscular reflexes of the upper airway) and predispose the child to obstructed breathing during sleep (7). Adenotonsillectomy (AT), the first-line therapy recommended for most children by the American Academy of Pediatrics, has been associated with an improvement in behavior, quality of life, and polysomnography parameters (7–9). Even after the performance of a successful AT, OSA persists in a substantial proportion of children, which implies that other risk factors such as craniofacial skeletal abnormalities and childhood obesity are also critical in the development and progression of OSA (6, 10, 11).

Lateral cephalometry radiography remains a suitable screening tool to evaluate the adenoidal tissue size, craniofacial skeletal characteristics, and the site of airway obstruction in children with OSA (12). Cephalometric studies have shown that adenotonsillar hypertrophy and decreased pharyngeal diameters at the levels of the adenoids were highly prevalent in the OSA group (13). Certain craniofacial skeletal abnormalities have been linked to OSA in children, such as maxillary and mandibular retrognathia, maxillary transverse constriction, increased mandibular plane angle, and inferiorly positioned hyoid bone (14–16). Reduced SNB (sella-nasion-B point) angle and increased ANB (A point-nasion-B point) angle were described among children with OSA (15). The development of the cranial base influences the growth of the head and face regions. A shorter cranial base has been associated with a vertical growth patten and may play a role in OSA in children (17). However, some other contradictory studies do not support such relationships (18, 19). At present, there is insufficient evidence to the effect that craniofacial morphology is or is not associated with OSA (20). Identification of craniofacial features that may be associated with OSA in children is important since these features are routinely evaluated in dental and orthodontic practices and may help in the screening, diagnosis, and future management of OSA in children.

Therefore, the aim of this study is to identify cephalometric variables associated with the presence and severity of OSA in children.

## Materials and methods

### Study design and samples

A total of 82 children with OSA and 77 controls were included in this observational prospective study. This study was approved by the Medical Ethics Committee of Stomatology Hospital of Shandong University (Approval No. 20210405) and the Ethics Committee of Ningbo Stomatology Hospital (Approval No. 330202022-202100001). Informed consent was obtained from the parents of all participants.

Children with OSA were transferred from the Ningbo ENT hospital. Patients were eligible for inclusion if they were 5–10 years of age, diagnosed with OSA on the basis of polysomnography, and if they underwent standardized cephalograms. Patients were excluded if they had craniofacial or growth abnormalities and a history of orthodontic treatment, tonsillectomy, or adenoidectomy. Control subjects were consecutively recruited from first-visit patients in Stomatology Hospital of Shandong University and Ningbo Stomatology Hospital. The exclusion criteria of the control subjects were the presence of snoring and congenital disease. Children were included as control subjects if they were 5–10 years of age, if they underwent standardized cephalograms, and if they were at low risk on the Pediatric Sleep Questionnaire (PSQ). The included samples were divided into two groups according to their age: 5–7 age group (28 OSA children and 30 controls) and 8–10 age group (54 OSA children and 47 controls).

### Pediatric Sleep Questionnaire

All 82 OSA children included in this study underwent an overnight PSG recording (SOMNOscreenTM Plus PSG, Randersacker, Germany) at the Ningbo ENT hospital. PSG allows a recording of thoracic and abdominal movements, airflow by nasal cannula, pulse oximetry, electroencephalogram, body position, electrooculograms, leg and chin electromyograms, and an electrocardiogram. The respiratory scoring rules were based on the American Academy of Sleep Medicine manual (21). Subjects with an obstructive apnea–hypopnea index (OAHI) score ≥1 time/h were defined as having OSA, and the severity of OSA was also indexed by using the OAHI score (22).

### Questionnaire

To evaluate the risk of OSA, each parent of the control subjects was asked to complete a translated and validated PSQ. The PSQ was specifically developed to calculate the risk of OSA in children with high reliability and good validity (23). This questionnaire consists of four sections with a total of 22 questions focused on three symptoms: snoring, excessive daytime sleepiness, and inattentive/hyperactive behavior (23). Subjects with less than eight positive answers were considered at low risk or no risk for OSA and were regarded as controls in this study.

### Cephalogram analysis

**Figure 1:**
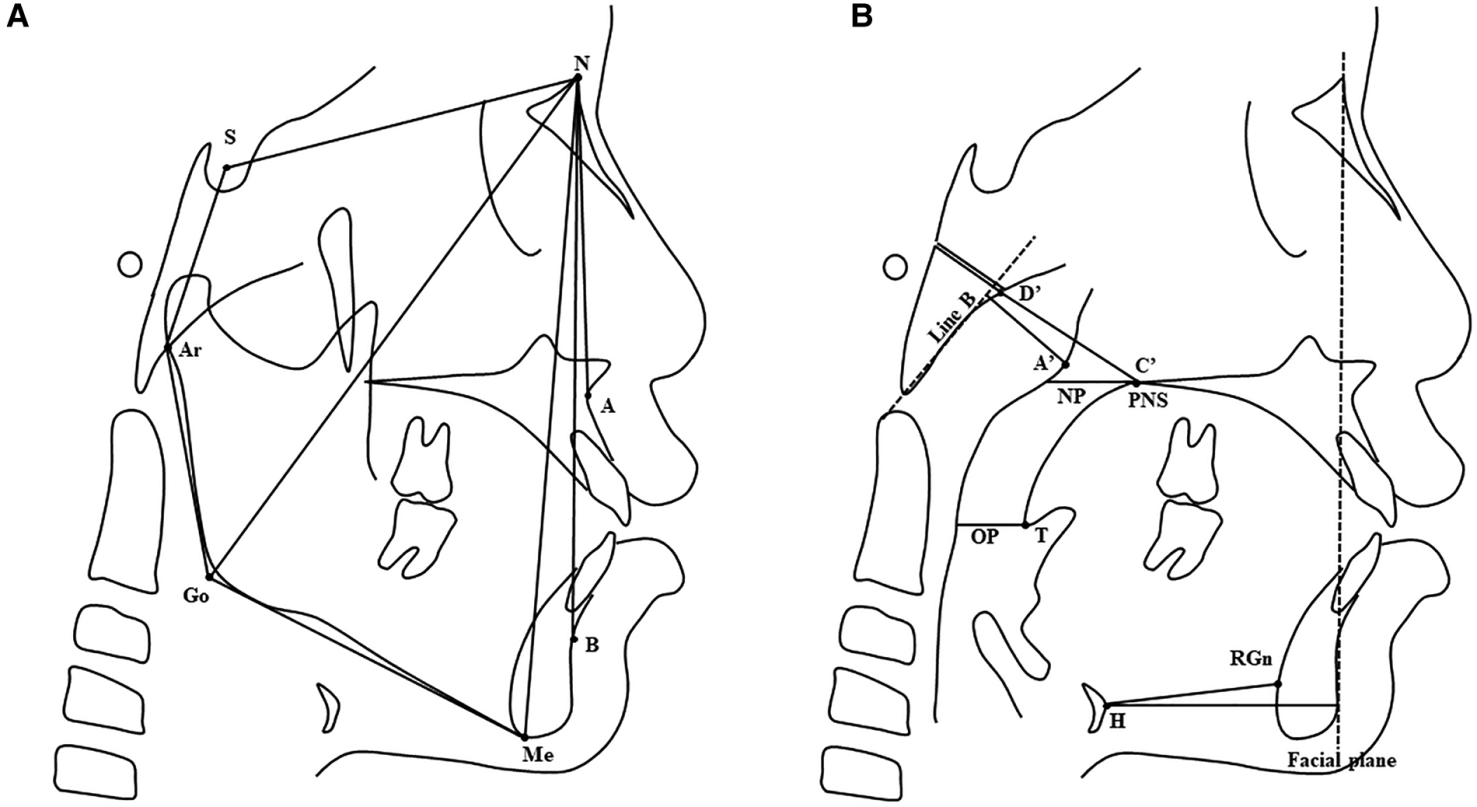
(**A**) Craniofacial skeletal variables. Landmarks: S, sella; N, nasion; A, A point; B, B point; Ar, articulare; Go, gonion; Me, menton. (**B**) Upper airway, adenoid, and hyoid bone variables. Landmarks: PNS, posterior nasal spine; T, tip of soft palate; A', A' point (maximal convexity along the inferior margin of adenoid shadow); C’, C’ point (posterior nasal spine); D', D' point (anteroinferior edge of phenobasioccipital synchondrosis); H, hyoid; RGn, retrognathion. Reference line: Line B, the line drawn along the straight part of the anterior margin of the basiocciput; Facial plane, the line formed by the nasion and pogonion. Variables: NP, nasopharyngeal airway space; OP, oropharyngeal airway space. See Table 1 for a detailed description of cephalometric variables.

All included participants underwent cephalometric radiography with the same digital x-ray unit (ORTHOPHOS XG 3D ready Ceph, Sirona Dental Systems GmbH, Bensheim, Germany). They were in an upright position with their teeth in centric occlusion and their head in natural position. At the time of image analysis, the operator was blinded to the clinical information of the subjects. Cephalometric variables are defined in Table 1 and shown in Figure 1. Cephalometric measurements were repeated on 20 randomly chosen images by an investigator (QX) with a 2-week time interval and were also repeated by another investigator (XW).

**Table 1:** Definitions of cephalometric variables.

Variable	Definition
**Craniofacial skeletal variables**	
SNA	Antero-posterior position of the maxilla relative to the anterior cranial base.
SNB	Antero-posterior position of the mandible relative to the anterior cranial base.
ANB	Antero-posterior relation of the maxilla and the mandible.
S-N	Distance between the nasion and the sella, which represents the anterior cranial base length.
S-Ar	Distance between the sella and the articulare, which represents the posterior cranial base length.
Ar-Go	Distance between the articulare and the gonion, which represents the ramus height.
Go-Me	Distance between the gonion and the menton, which represents the body of mandible length.
SA	Saddle angle, angle formed between nasion–sella–articulare.
AA	Articular angle, angle formed between sella–articulare–gonion.
GA	Gonial angle, angle formed between articulare–gonion–menton.
LGA	Lower gonial angle, the angle formed between nasion–menton–gonion.
Sum	Total angle, the sum of the saddle angle, articular angle, and gonial angle.
**Upper airway, adenoid, and hyoid bone variables**	
NP	Nasopharyngeal airway space, anteroposterior distance between the posterior pharyngeal wall and the posterior nasal spine (along the parallel line to the Frankfort horizontal plane).
OP	Oropharyngeal airway space, anteroposterior distance between the posterior pharyngeal wall and the tip of the uvula (along the parallel line to the Frankfort horizontal plane).
A	Distance between A' point (maximal convexity along the inferior margin of the adenoid shadow) to line B (drawn along the straight part of the anterior margin of the basiocciput), which represents the absolute value of the adenoid.
N	Distance between C' point (the posterior superior edge of the hard palate) and D' point (the anteroinferior edge of phenobasioccipital synchondrosis), which represents the linear dimensions of the bony nasopharynx.
A/N	Ratio of A and N, which represents the relative size of the adenoid.
H-RGn	The distance from the hyoid to the retrognathion.
H-FP	Anteroposterior distance between the hyoid and the facial plane (line formed by the nasion and the pogonion).

### Statistical analysis

All data were statistically analyzed using SPSS software (version 26.0, Chicago, III). The measurement error was determined by using Dahlberg's formula, which ranged between 0.581 and 1.441 mm for linear measurements and between 0.601° and 0.778° for angular measurements. An intraclass correlation coefficient (ICC) was used for determining the interobserver reliability and intraobserver reliability of the measurements. The ICC ranged from 0.891 to 0.979, showing repeated agreement with regard to all measurements.

Whether the data are normally distributed was examined by using the Shapiro–Wilk test. The independent *t*-test (for normally distributed variables), non-parametric Mann–Whitney test (for non-normally distributed variables), and *χ*^2^ test (for categorical variables) were used to compare the differences in the demographic characteristics between OSA children and the controls in different age groups. Demographic characteristics that were significantly different between OSA children and the controls were used as covariate(s) in the following analysis. One-way multivariate analysis of covariance was used to compare the differences of cephalometric variables between OSA children and control subjects.

Hierarchical regression analysis was performed to examine the relationship between cephalometric variables and OSA severity (expressed by OAHI) in different age groups. In step 1, since the BMI z-score was reported as a significant correlation factor with OSA severity (24), we included it in the model as a control variable to exclude its confounding effect. Then, the A/N ratio was entered into the model in step 2 to calculate its explanatory power in OSA severity. In step 3, we added each cephalometric variable (except for A/N) individually and ran a series of models. Cephalometric variables significantly correlated with OAHI (*P *< 0.05) after controlling for the BMI z-score, and the A/N ratio was included in the “final” multivariate model.

## Results

There was no significance in age, gender, and BMI z-score between OSA children and controls in the 5–7 age group (Table 2). In the 8–10 age group, OSA children and controls approximately matched in age and gender, while OSA children had a higher BMI z-score than controls (Table 1). Therefore, the BMI z-score was entered as a covariate in the comparison of cephalometric variables between OSA children and controls in the 8–10 age group.

**Table 2:** The included samples’ demographic and clinical characteristics.

Variable	5–7 age (mean ± SD)		*P*-value	8–10 age (mean ± SD)		*P*-value
	Control (30^a^)	OSA (28^a^)		Control (47^a^)	OSA (54^a^)	
Age (years)	6.24 ± 0.83	6.14 ± 0.88	0.695	8.82 ± 0.85	8.87 ± 0.92	0.823
Gender	40% (F)	68% (F)	0.534	43% (F)	44% (F)	0.848
BMI z-score	−0.04 ± 1.17	0.29 ± 1.03	0.585	−0.17 ± 0.83	0.92 ± 0.20	0.005*
OAHI (times/h)	–	5.49 ± 3.49	–	–	5.59 ± 5.02	–

OSA, obstructive apnea–hypopnea; OAHI, obstructive apnea–hypopnea index; F, female.

^a^ Number of samples.

*Statistically significant (*P *< 0.05).

In the 5–7 age group, the SNB angle, nasopharynx (NP), oropharynx (OP), and bony nasopharynx (N) of OSA children were all significantly smaller than those of control subjects, and increased adenoid size (A) and A/N ratio were found among OSA children (Table 3). In the 8–10 age group, the SNA angle, SNB angle, and OP were smaller, and the A/N ratio was larger among OSA children compared with controls. In addition, the ramus height (Ar-Go) of OSA children was significantly smaller than that of controls. There was no significant difference in the hyoid position (H-RGn and H-FP) between OSA children and controls in both age groups (Table 3).

**Table 3:** Comparison of the cephalometric variables between OSA children and control subjects in different age groups.

Variables	5–7 age (mean ± SD)		*P-*value	8–10 age^a^ (mean ± SD)		*P-*value
	Control	OSA		Control	OSA	
**Craniofacial skeletal variables**						
SNA (°)	81.1 ± 3.2	79.2 ± 4.1	0.089	82.2 ± 1.1	79.7 ± 0.5	0.042*
SNB (°)	78.2 ± 3.3	75.2 ± 3.9	0.007*	78.1 ± 1.2	75.3 ± 0.5	0.046*
ANB (°)	3.4 ± 3.1	4.1 ± 4.3	0.569	3.9 ± 1.2	34.2 ± 0.5	0.816
S-N (mm)	58.6 ± 2.6	59.6 ± 2.6	0.161	59.0 ± 1.0	60.4 ± 0.4	0.240
S-Ar (mm)	26.9 ± 3.1	27.7 ± 3.1	0.395	27.9 ± 1.8	30.7 ± 0.7	0.151
Ar-Go (mm)	37.6 ± 3.1	36.3 ± 4.1	0.247	40.5 ± 1.6	37.7 ± 0.7	0.042*
Go-Me (mm)	54.9 ± 3.8	54.0 ± 6.7	0.572	56.5 ± 0.2	58.5 ± 0.8	0.391
SA (°)	120.6 ± 5.4	122.3 ± 4.2	0.222	121.6 ± 1.7	122.4 ± 0.7	0.674
AA (°)	152.2 ± 6.4	151.9 ± 5.5	0.849	151.6 ± 2.2	151.7 ± 0.9	0.968
GA (°)	124.4 ± 7.8	123.2 ± 6.4	0.540	125.0 ± 2.1	126.2 ± 0.9	0.597
LGA (°)	77.7 ± 4.5	76.0 ± 5.4	0.245	79.0 ± 1.6	78.7 ± 0.7	0.890
Sum (°)	397.3 ± 5.4	397.4 ± 5.1	0.945	398.1 ± 1.7	400.3 ± 0.7	0.266
**Upper airway, adenoid, and hyoid bone variables**						
NP (mm)	19.4 ± 2.7	15.4 ± 4.4	<0.001*	21.2 ± 1.9	18.2 ± 0.8	0.165
OP (mm)	15.2 ± 2.1	10.4 ± 2.5	<0.001*	15.2 ± 1.4	10.3 ± 0.6	0.003*
A (mm)	9.8 ± 1.1	12.0 ± 3.5	0.010*	11.2 ± 1.1	11.7 ± 0.5	0.667
N (mm)	22.0 ± 2.0	18.5 ± 2.8	<0.001*	23.8 ± 1.0	21.8 ± 0.4	0.091
A/N	0.45 ± 0.05	0.65 ± 0.14	<0.001*	0.50 ± 0.04	0.59 ± 0.02	0.044*
H-RGn (mm)	25.2 ± 4.2	24.9 ± 4.4	0.844	28.2 ± 1.4	26.8 ± 0.6	0.371
H-FP (mm)	35.3 ± 4.4	35.3 ± 4.2	0.985	38.7 ± 1.5	37.0 ± 0.6	0.321

OSA, obstructive sleep apnea. See Table 1 for detailed definitions of each cephalometric variable.

^a^ With the BMI z-score as a covariate in the 8–10 age group.

*Statistically significant (*P* < 0.05).

The results of hierarchical regression analysis of the 5–7 age group showed that the BMI z-score was not significantly correlated with the OAHI (Δ*R*^2 ^= 0.010, *P *= 0.618) (Table 4). Given the widely accepted influence of obesity on the OAHI, we deemed it appropriate to include the BMI z-score in the following models. The A/N ratio accounted for 40.0% of the variance in the OAHI [*F*_(2,25)_ = 8.679, *P *= 0.001]. After controlling for the BMI z-score and A/N, it was found that the gonial angle (GA) and lower gonial angle (LGA) had a significant correlation with the OAHI (Table 4). Due to a strong correlation between GA and LGA (*r *= 0.775, *P *< 0.001), we chose LGA with more clinical significance to include it in the final multivariate model. The final model consisted of the BMI z-score, and A/N and LGA accounted for 55.6% and 50.1% (expressed by *R*^2^ and adjusted *R*^2^, respectively) of the variation in the OAHI [*F*_(3,24)_ = 10.031, *P *< 0.001] (Table 5).

**Table 4:** Hierarchical regression analysis, with the BMI z-score in step 1, A/N ratio in step 2, and each of the 16 cephalometric variables individually in step 3, used to predict the OAHI in the 5–7 age group.

	Variables in each regression block	Significant individual predictor variables with standardized coefficient and *p*-value	Model *R*^2^; Δ*R*^2^; *P-*value
Step 1	BMI z	–	0.010; 0.010; 0.618
Step 2	BMI z, A/N	A/N (0.636, <0.001)	0.410; 0.363*; <0.001
Step 3			
Model 1	BMI z, A/N, SNA	A/N (0.650, <0.001)	0.474; 0.408; 0.101
Model 2	BMI z, A/N, SNB	A/N (0.653, <0.001)	0.442; 0.037; 0.248
Model 3	BMI z, A/N, ANB	A/N (0.634, <0.001)	0.413; 0.004; 0.708
Model 4	BMI z, A/N, S-N	A/N (0.662, <0.001)	0.428; 0.019; 0.385
Model 5	BMI z, A/N, S-Ar	A/N (0.691, <0.001)	0.421; 0.038; 0.122
Model 6	BMI z, A/N, Ar-Go	A/N (0.634, <0.001)	0.412; 0.002; 0.763
Model 7	BMI z, A/N, Go-Me	A/N (0.701, 0.001)	0.436; 0.026; 0.301
Model 8	BMI z, A/N, SA	A/N (0.645, <0.001)	0.419; 0.010; 0.534
Model 9	BMI z, A/N, AA	A/N (0.644, <0.001)	0.423; 0.013; 0.463
Model 10	BMI z, A/N, GA	A/N (0.699, <0.001), GA (0.416, 0.009)	0.558; 0.148*; 0.009
Model 11	BMI z, A/N, LGA	A/N (0.663, <0.001), LGA (0.387, 0.010)	0.556; 0.147*; 0.010
Model 12	BMI z, A/N, Sum	A/N (0.650, <0.001)	0.473; 0.063; 0.103
Model 13	BMI z, A/N, NP	A/N (0.522, 0.003)	0.480; 0.071; 0.084
Model 14	BMI z, A/N, OP	A/N (0.679, <0.001)	0.436; 0.026; 0.299
Model 15	BMI z, A/N, H-RGn	A/N (0.653, <0.001)	0.424; 0.014; 0.447
Model 16	BMI z, A/N, H-FP	A/N (0.644, <0.001)	0.414; 0.004; 0.686

BMI z, BMI z-score; OAHI, obstructive apnea-hypopnea index. See Table 1 for detailed definitions of each cephalometric variable.

*Statistically significant (*P *< 0.05).

**Table 5:** Final hierarchical regression analysis with the BMI z-score in step 1, A/N ratio in step 2, and other significant cephalometric variables in step 3, used to predict the OAHI in the 5–7 age group.

		*B*	SE	Beta	*t*	*P*	Collinearity statistics	
							Tolerance	VIF
Step 1	BMI z	0.333	0.66	0.098	0.505	0.618	1	1
Step 2	BMI z	0.096	0.523	0.028	0.183	0.856	0.988	1.012
	A/N	16.314	3.963	0.636	4.117	<0.001	0.988	1.012
Step 3	BMI z	0.260	0.467	0.077	0.558	0.582	0.972	1.028
	A/N	17.005	3.515	0.663	4.837	<0.001*	0.983	1.017
	LGA	0.250	0.089	0.387	2.815	0.010*	0.978	1.023
Step 1: *R*^2^ = 0.010, adjusted *R*^2^ = −0.028, Δ*R*^2^ = 0.010								
Step 2: *R*^2^ = 0.410, adjusted *R*^2^ = 0.363, Δ*R*^2^ = 0.400*								
Step 3: *R*^2^ = 0.556, adjusted *R*^2^ = 0.501, Δ*R*^2^ = 0.147*								

BMI z, BMI z-score; OAHI, obstructive apnea–hypopnea index; *B*, unstandardized regression coefficient; SE, standard error; Beta, standardized coefficient; *t*, *t*-value; VIF, variance inflation factor.

*Statistically significant (*P *< 0.05). See Table 1 for detailed definitions of each cephalometric variable.

The results of the hierarchical regression analysis of the 8–10 age group are shown in Table 6. The BMI z-score accounted for 25.2% of the variance in the OAHI [*F*_(1,51)_ = 20.441, *P *< 0.001] and the A/N ratio 6.6% of the variance in the OAHI [*F*_(2,50)_ = 10.722, *P *< 0.001]. After controlling for the BMI z-score and A/N, it was found that LGA, H-RGn, and H-NP had a significant correlation with the OAHI. A strong correlation was present between H-RGn and H-NP (*r *= 0.821, *P *< 0.001), since both revealed the hyoid position. By comparing the value of ΔR^2^, H-RGn was chosen to be included in the final multivariate model. The final model consisted of the BMI z-score, A/N, LGA, and H-RGn, and accounted for 50.9% and 46.8% (expressed by *R*^2^ and adjusted *R*^2^, respectively) of the variation in the OAHI [*F*_(4, 48)_ = 10.307, *P *< 0.001] (Table 7).

**Table 6:** Hierarchical regression analysis, with the BMI z-score in step 1, A/N ratio in step 2, and each of the 16 cephalometric variables individually in step 3, used to predict the OAHI in the 8–10 age group.

	Variables in each regression block	Significant individual predictor variables with standardized coefficient and *P*-value	Model *R*^2^; Δ*R*^2^, *P*-value
Step 1	BMI z	BMI z (0.502, <0.001)	0.252; 0.252*; <0.001
Step 2	BMI z, A/N	BMI z (0.362, 0.009), A/N (0.292, 0.033)	0.317; 0.066*; 0.033
Step 3			
Model 1	BMI z, A/N, SNA	BMI z (0.364, 0.010), A/N (0.291, 0.035)	0.318; <0.001; 0.909
Model 2	BMI z, A/N, SNB	BMI z (0.337, 0.017), A/N (0.326, 0.022)	0.289; 0.012; 0.347
Model 3	BMI z, A/N, ANB	BMI z (0.348, 0.013), A/N (0.312, 0.028)	0.323; 0.005; 0.539
Model 4	BMI z, A/N, S-N	BMI z (0.362, 0.010), A/N (0.292, 0.035)	0.317; <0.001; 0.999
Model 5	BMI z, A/N, S-Ar	BMI z (0.334, 0.014), A/N (0.290, 0.031)	0.358; 0.041; 0.082
Model 6	BMI z, A/N, Ar-Go	BMI z (0.267, 0.051), A/N (0.310, 0.019)	0.349; 0.070; 0.022
Model 7	BMI z, A/N, Go-Me	BMI z (0.348, 0.012), A/N (0.319, 0.022)	0.335; 0.017; 0.264
Model 8	BMI z, A/N, SA	BMI z (0.340, 0.015), A/N (0.299, 0.030)	0.331; 0.014; 0.314
Model 9	BMI z, A/N, AA	BMI z (0.352, 0.011), A/N (0.266, 0.053)	0.338; 0.020; 0.226
Model 10	BMI z, A/N, GA	BMI z (0.347, 0.012), A/N (0.341, 0.020)	0.330; 0.013; 0.334
Model 11	BMI z, A/N, LGA	BMI z (0.314, 0.019), A/N (0.360, 0.008), LGA (0.266, 0.025)	0.4384; 0.067*; 0.025
Model 12	BMI z, A/N, Sum	BMI z (0.350, 0.010), A/N (0.331, 0.015)	0.361; 0.043; 0.074
Model 13	BMI z, A/N, NP	BMI z (0.350, 0.013), A/N (0.309, 0.029)	0.321; 0.004; 0.588
Model 14	BMI z, A/N, OP	BMI z (0.363, 0.008), A/N (0.249, 0.066)	0.360; 0.042; 0.078
Model 15	BMI z, A/N, H-RGn	BMI z (0.329, 0.009), A/N (0.313, 0.013), H-RGn (0.358, 0.002)	0.445; 0.127*; 0.002
Model 16	BMI z, A/N, H-FP	BMI z (0.347, 0.007), A/N (0.327, 0.011), H-NP (0.330, 0.004)	0.425; 0.108*; 0.004

BMI z, BMI z-score; OAHI, obstructive apnea–hypopnea index. See Table 1 for detailed definitions of each cephalometric variable.

*Statistically significant (*P *< 0.05).

**Table 7:** Final hierarchical regression analysis with the BMI z-score in step 1, A/N ratio in step 2, and other significant cephalometric variables in step 3, used to predict the OAHI in the 8–10 age group.

		*B*	SE	Beta	*t*	*P*	Collinearity statistics	
							Tolerance	VIF
Step 1	BMI z	0.107	0.026	0.502	4.141	<0.001*	1	1
Step 2	BMI z	0.077	0.028	0.362	2.722	0.009*	0.772	1.296
	A/N	12.481	5.689	0.292	2.194	0.033*	0.772	1.296
Step 3	BMI z	0.06	0.025	0.282	2.406	0.020*	0.747	1.339
	A/N	16.197	5.061	0.379	3.212	0.002*	0.731	1.368
	H-RGn	0.422	0.121	0.354	3.484	0.001*	0.993	1.007
	LGA	0.269	0.108	0.26	2.496	0.016*	0.946	1.057
Step 1: *R*^2^ = 0.252, adjusted *R*^2^ = 0.237, Δ*R*^2^ = 0.252*								
Step 2: *R*^2^ = 0.317, adjusted *R*^2^ = 0.290, Δ*R*^2^ = 0.066*								
Step 3: *R*^2^ = 0.509, adjusted *R*^2^ = 0.468, Δ*R*^2^ = 0.191*								

BMI z, BMI z-score; OAHI, obstructive apnea–hypopnea index; *B*, unstandardized regression coefficient; SE, standard error; Beta, standardized coefficient; *t*, *t*-value; VIF, variance inflation factor.

*Statistically significant (*P *< 0.05). See Table 1 for detailed definitions of each cephalometric variable.

## Discussion

In this study, the cephalometric variables identified were associated with the presence and severity of OSA in children. OSA occurs in children of all ages. In our study, we focused our attention on children aged between 5 and 10 years, who are the main population group for early orthodontic treatment. The peak prevalence of OSA in children occurred in the preschool age group (at the age of 6–7), in which the increased adenotonsillar growth reached its maximum size in relation to the upper airway (25, 26). Therefore, taking into consideration the variability of OSA in developmental age (27, 28), we divided the samples according to age into two age groups (5–7 age group and 8–10 age group).

It was not surprising to find an increased A/N ratio among OSA children in the 5–7 age group. The lymphoid tissue of Waldeyer’s ring develops at a higher rate between 3 and 7 years of age and is more susceptible to recurrent respiratory infections due to immature immune function (29, 30). In addition, it was noted that children with OSA had a smaller bony nasopharynx compared with controls. According to the theory proposed by Guilleminault and colleagues, adenoids were entrapped in a relatively small space, which may be the inciting event triggering mouth breathing and the eventual adenoid growth (31, 32). Due to the mismatching of soft tissue and skeletal structure, the nasopharynx space (at the level of the posterior nasal spine) was smaller among children with OSA. In this study, the SNB angle of OSA children was significantly smaller than that of controls, which was consistent with the results of previous studies. Deng and Gao found that the SNB angle of OSA children (mean 75.82, SD 4.30) was smaller than that in the control group (mean 78.71, SD 2.61) (14). Lee et al. reported that preschool children with OSA presented a skeletal class II pattern with a retropositioned mandible (33). The mandibular retrognathism may contribute to the narrowing of the oropharynx (also observed among OSA children in this study) and subsequently to the susceptibility to OSA development.

In the 8–10 age group, although the A/N ratio of OSA children was still larger than that of controls, there were no significant differences between OSA children and controls in terms of the adenoid size (A) and the bony nasopharynx (N), due to the spontaneous remission of hypertrophied adenoid tissues and the development of the nasomaxillary complex. The difference in the nasopharynx space (at the level of the posterior nasal spine) was less distinct between OSA children and controls in the 8–10 age group, compared with that in the 5–7 age group. A reduced SNB angle and narrowing oropharynx were also observed among OSA children in the 8–10 age group. In addition, OSA children in the 8–10 age group had a significantly smaller SNA angle. It was noteworthy that OSA children in the 8–10 age group had a significantly shorten ramus height (Ar-Go). The growth of the ramus height is mainly determined by the new bone deposition on the mandibular condyle, which involves highly complicated environmental and genetic factors (34, 35). The insufficient growth of the mandibular ramus is usually associated with a reduced posterior facial height, divergent growth pattern, and skeletal class II relationship (36, 37), whereas significant condylar and ramus growth could prevent backward rotation of the mandible (38).

Due to the significant correlation with OSA severity as reported (24), the BMI z-score was included in the model in step 1 as a control variable. In the 5–7 age group, the BMI z-score was not significantly correlated with OSA severity, whereas the A/N ratio accounted for 40.0% of the variance in the OAHI, suggesting that adenoid hypertrophy was the main cause of OSA in children. Several studies have investigated the relationship between adenoid size and OSA severity. Brooks et al. evaluated 33 OSA children with a mean age of 4.5 years and found that the A/N ratio correlated with the duration of obstructive apneas more than the number of obstructive apneas (39). Jain and Sahni reported a significant correlation between adenoid size and OSA severity by evaluating 40 OSA children aged between 4 and 12 years (40). In this study, the results highlighted the direct etiologic role played by adenoid hypertrophy in the OSA severity of the 5–7 age group and suggested that adenoidectomy may be a priority treatment strategy at this stage of age.

After controlling for the BMI z-score and A/N ratio, LGA was a significant predictor of OSA severity in the 5–7 age group, accounting for 14.7% of the variance in the OAHI. Correlative data between craniofacial skeletal variables and OSA severity in pediatric OSA are limited, especially taking into account the effect of potential confounders. Some investigators have suggested that as adenoid hypertrophy blocks the upper airway, children resort to mouth breathing, resulting in an “adenoid face” such as a steep mandibular plane and a retrusive-tending chin. The supporting evidence indicated that adenoidectomy was associated with an acceleration of mandibular growth and correction of the craniofacial growth pattern (35, 41). However, evidence also shows that children who underwent adenotonsillectomy maintained their original craniofacial abnormalities (26, 42, 43). In this study, LGA was still correlated with OSA severity after controlling for the BMI z-score and A/N ratio, which suggested that the growth pattern played a significant role in the OSA severity in children. There may be other important developmental or genetic determinants that predispose children to abnormal growth patterns and then worsen OSA (26). Therefore, in addition to referral to otolaryngology, appropriate orthodontic treatment, including a correction of growth patterns to open airways, is necessary to alleviate OSA severity in children aged 5–7 years old.

Unlike in the 5–7 age group, the BMI z-score accounted for 25.2% of the variance in the OAHI, whereas the A/N ratio accounted for only 6.6% of the variance in the OAHI in the 8–10 age group. In other words, obesity replaced adenoid hypertrophy as the main factor influencing the severity of OSA in this stage of age. Lam et al. found that obese children had significantly higher AHI values than those of non-obese children and demonstrated a significant, although mild, correlation between OSA severity and the degree of obesity in OSA children ranging from 1 to 15 years old (24). In this study, we focused on OSA children aged 5–7 and 8–10 years separately and found that obesity had a significant and remarkable correlation with OSA severity in children aged 8–10 years. As adenoid hypertrophy plays a minor role in the severity of OSA, adenoidectomy should be considered with caution in this age group.

In addition to LGA, the hyoid position (H-RGn) significantly correlated with the OAHI in the 8–10 age group. The relationship between hyoid position and OSA severity has been reported in adults. Bilici et al. showed that the hyoid-menton distance between patients with severe OSA was longer than that in other OSA groups (44). Stipa et al. also described that the distance between the hyoid and the mandibular plane was a significant determinate in the model for OSA severity (45). However, there is limited literature reporting the relationship in children. In adults, the lower position of the hyoid may influence the tongue position and thus the upper airway patency, since the hyoid bone serves as an anchor for the tongue muscles (46). These alterations may also occur in OSA children aged 8–10 years.

The final model indicated that the increases in OAHI variance accounted for by the BMI z-score and cephalometric variables were equal to 50.1% in the 5–7 age group and 46.8% in the 8–10 age group, which were all statistically significant. The results revealed a significant relationship between cephalometric variables and OSA severity in children. However, the values of variance in the OAHI variables that were accounted for were not very high. It should be noted that pediatric OSA is a dynamic process resulting from a combination of upper airway structural and neuromotor abnormalities. In addition to soft tissue hypertrophy, obesity, and craniofacial disharmony, other risk factors such as an impaired neural response and abnormal central arousal mechanism were also involved in the pathology of pediatric OSA (25, 47). Considering the complex pathology of pediatric OSA, the management of OSA in children requires multidisciplinary collaboration involving the pediatric physician, otolaryngologist, and orthodontist.

Our study has several limitations. Firstly, the size of the tonsils plays an important role in the development and progression of OSA in children (48), the evaluation of which was not performed in the present study. Secondly, due to a relatively small sample size, we could not detect further cephalometric variables that may be relevant to OSA presence and severity. Also, in this study, we used OAHI ≥1 time/h as a cutoff value to determine OSA children, while some studies use different values (>1, >1.5, >2) (49, 50). The different cutoff values may influence the results. However, based on the recommendations of ICSD-3 and most researchers, we deemed it more appropriate to use the current cutoff value (51–55).

## Conclusions

Adenoid hypertrophy was a major factor associated with OSA in preschool children, whereas obesity replaced adenoid hypertrophy as the main contributor to OSA in late childhood. Several craniofacial skeletal variables such as the SNB angle, ramus height, lower gonial angle, and hyoid position, were also associated with the presence and/or severity of OSA, which could be used to help recognize children at a higher risk of developing OSA.

## Data Availability

The original contributions presented in the study are included in the article/supplementary material; further inquiries can be directed to the corresponding author/s.
