# Corrigendum: Craniofacial and upper airway morphological characteristics associated with the presence and severity of obstructive sleep apnea in Chinese children

**DOI:** 10.3389/fped.2023.1208674

**Published:** 2023-06-09

**Authors:** Qiuping Xu, Xiaoya Wang, Na Li, Ying Wang, Xin Xu, Jing Guo

**Affiliations:** ^1^ Engineering Laboratory for Biomaterials and Tissue Regeneration, Ningbo Stomatology Hospital, Ningbo, Zhejiang, China & Savaid Stomatology School of Hangzhou Medical College, Hangzhou, Zhejiang, China; ^2^ Department of Orthodontics, School and Hospital of Stomatology, Cheeloo College of Medicine, Shandong University & Shandong Key Laboratory of Oral Tissue Regeneration & Shandong Engineering Laboratory for Dental Materials and Oral Tissue Regeneration & Shandong Provincial Clinical Research Center for Oral Diseases, Jinan, China; ^3^ Department of Stomatology, Beijing Tongren Hospital, Capital Medical University, Beijing, China; ^4^ Department of Implantology, School and Hospital of Stomatology, Cheeloo College of Medicine, Shandong University & Shandong Key Laboratory of Oral Tissue Regeneration & Shandong Engineering Laboratory for Dental Materials and Oral Tissue Regeneration& Shandong Provincial Clinical Research Center for Oral Diseases, Jinan, China

**Keywords:** orthodontics &amp, dentofacial orthopedics, upper airway, pediatric obstructive sleep apnea, cephalometry, craniofacial abnormities

A Corrigendum on Craniofacial and upper airway morphological characteristics associated with the presence and severity of obstructive sleep apnea in Chinese children By Xu Q, Wang X, Li N, Wang Y, Xu X and Guo J. (2023) Front. Pediatr. 11:1124610. doi: 10.3389/fped.2023.1124610

In the published article, there was an error in affiliation 3. Instead of “Department of Implantology, School and Hospital of Stomatology, Cheeloo College of Medicine, Shandong University & Shandong Key Laboratory of Oral Tissue Regeneration & Shandong Engineering Laboratory for Dental Materials and Oral Tissue Regeneration”, it should be “Department of Implantology, School and Hospital of Stomatology, Cheeloo College of Medicine, Shandong University & Shandong Key Laboratory of Oral Tissue Regeneration & Shandong Engineering Laboratory for Dental Materials and Oral Tissue Regeneration& Shandong Provincial Clinical Research Center for Oral Diseases, Jinan, China”.

In the published article, there was an error in affiliation 1. Instead of “Department of Orthodontics, Ningbo Stomatology Hospital & Savaid Stomatology School, Hangzhou Medical College, Ningbo, China”, it should be “Engineering Laboratory for Biomaterials and Tissue Regeneration, Ningbo Stomatology Hospital, Ningbo, Zhejiang, China & Savaid Stomatology School of Hangzhou Medical College, Hangzhou, Zhejiang, China”.

In the published article, there was an error in the listing order of affiliations 1–4. Instead of “Qiuping Xu^1,2†^, Xiaoya Wang^1,3†^, Na Li^4^, Ying Wang^4^, Xin Xu^3^ and Jing Guo^1,4^*

^1^Department of Orthodontics, School and Hospital of Stomatology, Cheeloo College of Medicine, Shandong University & Shandong Key Laboratory of Oral Tissue Regeneration & Shandong Engineering Laboratory for Dental Materials and Oral Tissue Regeneration & Shandong Provincial Clinical Research Center for Oral Diseases, Jinan, China

^2^Department of Stomatology, Beijing Tongren Hospital, Capital Medical University, Beijing, China

^3^Department of Implantology, School and Hospital of Stomatology, Cheeloo College of Medicine, Shandong University & Shandong Key Laboratory of Oral Tissue Regeneration & Shandong Engineering Laboratory for Dental Materials and Oral Tissue Regeneration& Shandong Provincial Clinical Research Center for Oral Diseases, Jinan, China

^4^Department of Orthodontics, Ningbo Stomatology Hospital & Savaid Stomatology School, Hangzhou Medical College, Ningbo, China”

It should be “Qiuping Xu^1,2,3†^, Xiaoya Wang^1,2,4†^, Na Li^1^, Ying Wang^1^, Xin Xu^4^ and Jing Guo^1,2^*

^1^Engineering Laboratory for Biomaterials and Tissue Regeneration, Ningbo Stomatology Hospital, Ningbo, Zhejiang, China & Savaid Stomatology School of Hangzhou Medical College, Hangzhou, Zhejiang, China

^2^Department of Orthodontics, School and Hospital of Stomatology, Cheeloo College of Medicine, Shandong University & Shandong Key Laboratory of Oral Tissue Regeneration & Shandong Engineering Laboratory for Dental Materials and Oral Tissue Regeneration & Shandong Provincial Clinical Research Center for Oral Diseases, Jinan, China

^3^Department of Stomatology, Beijing Tongren Hospital, Capital Medical University, Beijing, China

^4^Department of Implantology, School and Hospital of Stomatology, Cheeloo College of Medicine, Shandong University & Shandong Key Laboratory of Oral Tissue Regeneration & Shandong Engineering Laboratory for Dental Materials and Oral Tissue Regeneration& Shandong Provincial Clinical Research Center for Oral Diseases, Jinan, China”.

The authors apologize for this error and state that this does not change the scientific conclusions of the article in any way. The original article has been updated.

